# Association between tooth agenesis and developmental dental anomalies: an observational study with machine learning analysis

**DOI:** 10.1590/2177-6709.31.3.e2625183.oar

**Published:** 2026-08-07

**Authors:** Guilherme Henrique BORGES, Walbert A. VIEIRA, Isabela Vinhal HENRIQUES, Lígia Machado SILVA, Rubens SPIN-NETO, Marcos Alan Vieira BITTENCOURT, Luiz Renato PARANHOS

**Affiliations:** 1 Universidade Federal de Uberlândia, School of Dentistry (Uberlândia/MG, Brazil).; 2 Centro Universitário das Faculdades Associadas de Ensino, School of Dentistry (São João da Boa Vista/SP, Brazil).; 3 Aarhus University, Department of Dentistry and Oral Health - Division of Oral Radiology and Endodontics (Aarhus, Denmark).; 4 Universidade Federal da Bahia, School of Dentistry, Department of Social and Pediatric Dentistry (Salvador/BA, Brazil).; 5 Universidade Federal de Uberlândia, School of Dentistry, Department of Orthodontics (Uberlândia/MG, Brazil).

**Keywords:** Tooth agenesis, Dental anomalies, Machine learning, Cross-sectional study, Dental radiographs, Agenesia dental, Anomalias dentárias, Aprendizado de máquina, Estudo transversal, Radiografias dentárias

## Abstract

**Objective:**

To investigate the prevalence of tooth agenesis and its association with other developmental dental anomalies in non-syndromic patients. The study also evaluated the applicability of machine learning models to predict the occurrence of agenesis.

**Methods:**

This cross-sectional observational study analyzed 4,990 panoramic radiographs from radiology centers in Uberlândia, Minas Gerais/Brazil. It assessed the presence of tooth agenesis and associated anomalies, such as palatal displacement of canines, distoangulation of lower second premolars, tooth transposition, infraocclusion of deciduous molars, mesioangulation of lower second molars, and supernumerary teeth. The tooth agenesis code (TAC) mapped the patterns of occurrence. Fisher’s exact test, Student’s t-test, and logistic regression were used to analyze the data (*p* < 0.05). The machine learning models used included Decision Tree, Random Forest, XGBoost, Support Vector Machine, and Deep Learning.

**Results:**

Agenesis was found in 6.1% of patients, with a higher prevalence in females (57.6%) and younger individuals (mean age of 16.8 years). Dental transposition (OR: 19.11; *p* < 0.001), maxillary canine displacement (OR: 2.08; *p* < 0.001), and deciduous molar infraocclusion (OR: 147.73; *p* < 0.001) showed a significant association. The Random Forest model showed the best predictive performance, but with clinical limitations.

**Conclusion:**

Tooth agenesis is associated with an increased risk of other anomalies, highlighting the importance of early diagnosis and integrated therapeutic approaches.

## RESUMO

**Objetivo:**

Investigar a prevalência de agenesia dental e sua associação com outras anomalias dentárias de desenvolvimento em pacientes não sindrômicos. O estudo também avaliou a aplicabilidade de modelos de aprendizado de máquina para predizer a presença de agenesia.

**Métodos:**

Este estudo observacional transversal analisou 4.990 radiografias panorâmicas de centros de radiologia em Uberlândia, Minas Gerais. Avaliou a presença de agenesia dental e anomalias associadas, como deslocamento de caninos por palatino, distoangulação de segundos pré-molares inferiores, transposição dentária, infraoclusão de molares decíduos, mesioangulação de segundos molares inferiores e dentes supranumerários. O código de agenesia dentária (TAC) mapeou os padrões de ocorrência. Os testes exatos de Fisher, t de Student e regressão logística foram usados na análise dos dados (p < 0,05). Os modelos de aprendizado de máquina utilizados incluíram *Decision Tree, Random Forest, XGBoost, Support Vector Machine e Deep Learning.*

**Resultados:**

Agenesia foi encontrada em 6,1% dos pacientes, com maior prevalência em mulheres (57,6%) e indivíduos mais jovens (idade média de 16,8 anos). Transposição dentária (OR: 19,11; p < 0,001), deslocamento de canino por palatino (OR: 2,08; p < 0,001) e infraoclusão de molar decíduo (OR: 147,73; p < 0,001) apresentaram associação significativa. O modelo *Random Forest* apresentou o melhor desempenho preditivo, mas com limitações clínicas.

**Conclusões:**

Agenesia dental está associada a um maior risco de outras anomalias, destacando a relevância do diagnóstico precoce e de abordagens terapêuticas integradas.

## INTRODUCTION

Tooth agenesis is an anomaly characterized by the absence of teeth due to complex interactions between genetic, epigenetic, and environmental factors^1^ in the morphodifferentiation phase during odontogenesis.^2^ Its presentation is syndromic, which involves other organs or tissues, or non-syndromic, which only affects dentition.^3^

The various clinical manifestations of tooth agenesis reflect this condition’s genetic and phenotypic heterogeneity.^4^ Among them, the primary mutations occur in genes *AXIN2, EDA, LRP6, MSX1, PAX9, WNT10A,* and *WNT10B*. These genes appear isolated in individuals or families affected by agenesis.^5^ Tooth agenesis prevalence varies between 0.3% and 36.5%, according to geographical location, dental group, dentition, and ethnicity, and it is higher in the female sex.^6^ ^,^ ^7^

The literature has often highlighted the association between agenesis and other dental anomalies, such as transpositions,^8^ impaction,^9^ delayed dental development,^7^ ectopic eruption, supernumerary teeth, deciduous tooth retention, and other shape and size anomalies.^6^ ^,^ ^7^ ^,^ ^10^ That is possibly due to a genetic mutation that may cause different phenotypic expressions, i.e., different dental anomalies in the same individual might show distinct expressions of the same genetic code.^4^ This relationship between number, size, and shape anomalies suggests a typical genetic control with different phenotypic presentations.^10^

Developmental anomalies are associated with permanent tooth agenesis, decreasing overall tooth size and delaying dental development.^11^ ^,^ ^12^ Second premolar agenesis has also been associated with higher agenesis prevalence in other permanent teeth,^13^ upper lateral incisor microdontia,^4^ ^,^ ^14^ lower deciduous molar infraocclusion, palatally displaced upper canines,^15^ ^,^ ^16^ lower second premolar distal angulation, upper first molar ectopic eruption, and lower second premolar mesial angulation, among other combinations.^4^ ^,^ ^14^ ^,^ ^16^

Professionals attentive to early detection and management have focused on these associations among developmental anomalies, as they affect esthetics and occlusion.^2^ The relationship between the smaller upper lateral incisor crown and other teeth is among these associations.^7^ Another association might include the root morphology of incisors, which are more susceptible to root resorption during an occasional orthodontic treatment.^17^ ^,^ ^18^

Understanding patterns of tooth agenesis and their associations with other anomalies across different populations is essential for identifying variations within and between groups. This information is relevant not only for clinical practice, but also for phylogenetic and genetic studies, as most existing research remains descriptive, focusing mainly on the presence or absence of the condition.^10^ ^,^ ^17^ ^,^ ^19^ Patients presenting specific patterns of dental anomalies - such as tooth agenesis associated with ectopic eruptions and transpositions - require early orthodontic intervention to guide eruption of permanent teeth, prevent space loss, and reduce orthodontic treatment complexity.^20^

This study evaluated the prevalence of tooth agenesis in the analyzed population and investigated its associations with other developmental dental anomalies. Additionally, it examined the applicability of artificial intelligence through machine learning models to predict agenesis occurrence based on clinical and radiographic data. Demographic variables and anomaly patterns were incorporated into predictive algorithms to assess the potential of these technologies in supporting early diagnosis.

## METHODOLOGY

### ETHICAL CRITERIA

This study received approval from the Research Ethics Committee of a Higher Education Institution (Approval protocol #6.976.142). All individuals had their privacy rights respected. The manuscript was written according to the STROBE reporting guide for cross-sectional studies.^21^ The study did not use identification information from the participants.

### STUDY DESIGN

This was a cross-sectional observational study that used imaging exams to assess the outcomes of interest in two stages. The first stage verified the prevalence of dental anomalies. The second evaluated the association of agenesis with other dental anomalies.

### SAMPLE SELECTION

The sample originated from the analysis of panoramic radiographs as part of the dental treatment plan of individuals who attended radiological centers in Uberlândia/MG, Brazil, from October 19, 2021, to June 1, 2024.

The study included good diagnostic image quality radiographs of patients of both sexes aged between 8 and 30 years. The exclusion criteria were incomplete files (without a panoramic radiograph) and records of patients outside the study’s age group, submitted to or undergoing orthodontic treatment (diagnosed by the presence of brackets, retainers, or any other visible orthodontic piece in the radiographs, root resorption or remodeling possibly due to orthodontic treatment, and resin remnants suggesting orthodontic appliance or retainer use), and submitted to permanent tooth extraction (detected by the absence of dental elements, dental ridge integrity analysis, and alveolar bone healing). Craniofacial malformations and syndromes were identified through medical records provided by the radiological centers and the analysis of facial and intraoral photographs when included in the radiological documents.

Two previously trained professionals (one specialist in Orthodontics and Radiology and the other, specialist in Orthodontics) carefully analyzed all existing radiographic and photographic exams, following the same protocol and using the visual method. In case of doubt, a third professional (Orthodontics specialist) analyzed the exams, and the report was compared with those provided by the radiological centers attached to patients’ exams. The exams were re-evaluated in cases of diagnostic conflict. The study excluded the exams whose doubts remained unsolved. Panoramic radiographs were digitally analyzed in a dark room. Only 20 radiographs were evaluated daily, to prevent the operators’ visual fatigue.

**Figure 1:**
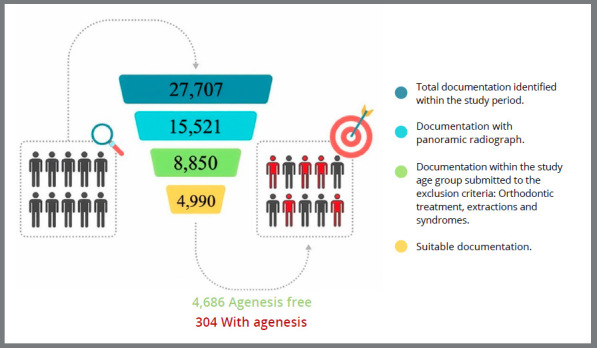
Schematic representation of convenience sample selection.

A total of 27,707 documents were identified, of which 8,850 fit the age group (8-30 years) and included panoramic radiographs among the radiographic exams. Moreover, 4,990 radiographic documents were eligible after removing duplicates. Figure 1 illustrates the convenience sample selection, for better visualization.

### DATA COLLECTION

The study investigated the prevalence of seven developmental dental anomalies usually described in the literature (tooth agenesis, palatal displacement of upper canines, lower second premolar distal angulation, dental transposition, deciduous molar infraocclusion, lower second molar mesial angulation, and supernumerary teeth)^10^, based on the selected panoramic radiographs.

The data found in Cfazmax software (A cloud-based online platform, accessible at *https://max.Cfaz.net*, Belo Horizonte, Minas Gerais, Brazil) were recorded in an Excel spreadsheet especially created for this study, containing patients’ registration numbers in the clinic; personal data, such as age and sex; exam date; exclusion criteria; the presence of agenesis; the presence of other developmental anomalies; and radiological exams when present (panoramic and periapical radiographs, profile cephalometric images, facial cone-beam computed tomography, and virtual models generated by dental arch scanning). It is worth noting that the sample did not include third molars, because of their unstable frequency and late formation.

### TOOTH AGENESIS DIAGNOSIS AND LABEL

Tooth agenesis diagnosis was based on the analysis of panoramic and periapical radiographs, medical records, and intraoral photographs when present. The method by Ericson and Kurol^22^ diagnosed the palatal displacement of upper canines by evaluating the relationship between the upper canine cusp tip position and the adjacent lateral incisor root. The method uses a diagram with reference lines and linear and angular measures to be traced in the panoramic radiograph, to analyze the higher or lower probability of canine impaction. Second premolars were analyzed considering their distal angle, following criteria by Shalish et al.,^23^ and using the angle measured between the lower second premolar long axis and the mandibular lower margin. Dental transposition identification was based on the position exchange of two adjacent teeth or tooth development or eruption in a position usually occupied by a non-adjacent tooth. Deciduous molar infraocclusion was diagnosed when the distance between the occlusal surface and plane was higher than 1 mm. Lower second molar mesial angulation was based on the initial blockage of the lower second molar eruption by the first adjacent molar. Finally, supernumerary teeth were diagnosed for teeth present beyond the normal dentition.

Each agenesis patient received an individual tooth agenesis label (i.e., a “code”, namely TAC) score.^24^ This label helps map tooth agenesis frequency in individuals, allowing an efficient and accurate dentition expression regarding the number and location of missing teeth.

### OPERATOR-DEPENDENT BIAS

The intra-examiner reproducibility assessment had the primary evaluator revisiting 30% of the sample (n = 1,497 randomly selected panoramic radiographs: www.random.org) and repeating the measurements 30 days after the primary analysis. The primary assessments (T1) were compared to those after 30 days (T2). A second evaluator analyzed the intra-examiner agreement. The second evaluator also measured 30% (n = 1,497) of panoramic radiographs. The analyses of the primary and secondary evaluators were compared. The intraclass correlation (ICC) and the obtained values (Kappa > 0.90) confirmed the assessments’ high reproducibility and reliability.

## DATA ANALYSIS

### STATISTICAL ANALYSIS

Stata software, version 18 (StataCorp LLC, College Station, TX, USA), hosted all analyses. The significance level was 5%.

Fisher’s exact test (categorical variables) and Student t-test (continuous variable) were used to analyze the sample according to sociodemographic characteristics (sex and age), describing it from absolute and relative frequencies (agenesis cases and anomaly distribution, number, and location). Besides descriptive analyses, anomaly prevalence was compared between patients with and without agenesis. Agenesis distribution was also compared according to the dental arch and homologous tooth.

Logistic regressions evaluated the associations between agenesis and anomalies and agenesis and shape anomalies. The association between agenesis and anomalies included the total study sample (n = 4,990). Crude and adjusted odds ratios were estimated for the sex and age of individuals.

### MACHINE LEARNING MODELS

The present study applied machine learning models to predict the presence or absence of tooth agenesis based on clinical and radiographic variables. The initial stage involved data preprocessing, ensuring their normalization and transformation into an adequate format for prediction algorithms. Input variables considered demographic and clinical information, including sex, age, and associated dental anomalies. The one-hot encoding method dichotomized all variables. This method generates a new variable with values from zero to one for each category. Agenesis presence was classified binarily (present or absent) as a response variable.

The sample was divided into two subsamples (70% training and 30% test). Considering the low tooth agenesis prevalence, the synthetic minority oversampling technique (SMOTE) balanced the training sample. Next, five machine learning models were produced: Decision Tree, Random Forest, XGBoost, SVM, and Deep Learning. A cross-validation (k = 10) adjusted the hyperparameters for the training set, to prevent overfitting.

After selecting the best hyperparameter of each model, the following metrics measured the predictive performance of algorithms: area under the curve (AUC), accuracy (ACC), sensitivity, specificity, positive predictive value (PPV), negative predictive value (NPV), R² value, and F1 score. Moreover, Shapley (SHAP) values were calculated for the best AUC model, to determine the relevance of each variable in predicting the study outcomes.^25^

The Python scikit-learn package was used to execute machine learning models.^26^

## RESULTS

### AGENESIS PREVALENCE

The study had 4,990 eligible patient documents, of which 304 (6.1%) reported agenesis. Female patients showed higher agenesis prevalence (*p* = 0.006) (Table 1). The average age of agenesis patients was significantly lower (*p* < 0.001).

**Table 1:** Sample distribution by sex and age according to agenesis presence.

	Total	Without agenesis n (%)	With agenesis n (%)	p-value
n	4,990 (100.0)	4,686 (93.9)	304 (6.1)	
Sex				0.006
Female	2,495 (50.0)	2,320 (49.5)	175 (57.6)	
Male	2,495 (50.0)	2,366 (50.5)	129 (42.4)	
Age - mean (SD)	18.3 (6.2)	18.4 (6.2)	16.8 (6.2)	< 0.001

Fisher’s exact test estimated the p-values for categorical variables, and Student t-test, for continuous variables.

### ASSOCIATION BETWEEN AGENESIS AND DENTAL ANOMALIES

**Figure 2:**
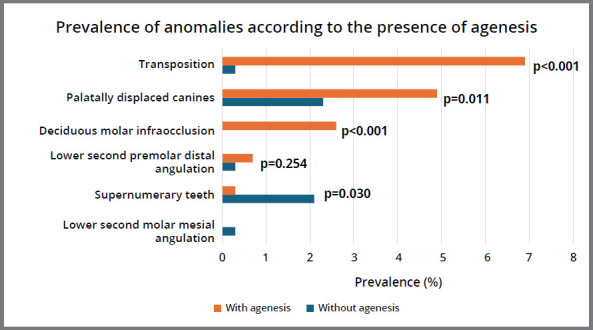
Comparison of the prevalence of the anomalies considered in this study, in patients with and without agenesis.

Figure 2 describes the prevalence of different anomalies according to agenesis presence. While transposition, deciduous molar infraocclusion, and palatally displaced canines prevalences were statistically higher in agenesis patients, supernumerary teeth prevalence was statistically higher in patients without agenesis. Lower second premolar distal angulation and lower second molar mesial angulation did not show statistically significant differences.

Around 15% of agenesis patients had some associated dental anomaly. Approximately 45% of agenesis cases were located in the lower arch and a just over 43%, in the upper arch. Around 43% of antimere teeth appeared on both sides. Dental transposition prevalence among the cases was around 7%, palatally displaced canines was 5%, and deciduous molar infraocclusion was 2.6%. The prevalence of other anomalies was lower than 1% (Table 2).

**Table 2:** Description of the anomalies considered in this study, and their distribution in agenesis patients.

	n (%)
Presence of anomaly	
No	259 (85.2)
Yes	45 (14.8)
Number of anomalies	
0	259 (85.2)
1	43 (14.1)
2	2 (0.7)
Arch	
Lower	136 (44.9)
Upper	131 (43.2)
Both	37 (11.9)
Homologous tooth	
Right	92 (30.4)
Left	81 (26.7)
Right/Left	131 (42.9)
Anomaly types	
Palatally displaced canines	15 (4.9)
Lower second premolar distal angulation	2 (0.7)
Transposition	21 (6.9)
Deciduous molar infraocclusion	8 (2.6)
Lower second molar mesial angulation	0 (0.0)
Supernumerary teeth	1 (0.3)

Table 3 presents the findings of crude and adjusted analyses for sex and age. The odds for palatally displaced canines were 2.08 times higher in agenesis patients than in those without the condition (*p* = 0.010). Similarly, the chances of transposition and infraocclusion were 19.11 and 147.73 times higher in agenesis patients than those without the condition. The high odds ratio values were due to the low prevalence of the analyzed outcomes.

**Table 3:** Crude and adjusted prevalence ratios of agenesis according to the identified anomalies.

	Crude		Adjusted	
	OR (95% CI)	p-value	OR (95% CI)	p-value
Anomaly types (n = 4,990)				
Palatally displaced canines	2.22 (1.27 - 3.86)	0.005	2.08 (1.19 - 3.63)	0.010
Lower second premolar distal angulation	2.21 (0.50 - 9.77)	0.296	1.65 (0.37 - 7.38)	0.509
Transposition	21.66 (11.18 - 41.96)	< 0.001	19.11 (9.78 - 37.33)	< 0.001
Deciduous molar infraocclusion	126.62 (15.78 - 1,015.74)	< 0.001	147.73 (18.23 - 1,196.76)	< 0.001
Lower second molar mesial angulation	-	-	-	-
Supernumerary teeth	0.16 (0.02 - 1.12)	0.065	0.17 (0.02 - 1.24)	0.080

Analyses adjusted for sex and age. No agenesis patient showed lower second molar mesial angulation, hindering the comparison.

### TOOTH AGENESIS LABEL

Each detected tooth agenesis received a label value (the TAC), which was counted by label repetition and described in Table 4. Thirty-four of the 304 tooth agenesis patients presented lower premolar agenesis on both lower arch sides (q3 (Left), q4 (Right)), followed by 33 individuals with upper lateral incisor agenesis affecting both upper arch sides (q1 (Right), q2 (Left)), 25 (q4 (Right)) and 24 (q3 (Left)) with unilateral agenesis in lower premolars, 21 (q1 (Right)) and 18 (q2 (Left)) with unilateral agenesis of upper lateral incisors, and so on in succession.

**Table 4:** Tooth agenesis code (TAC).

GENERAL TAC														
CASES	TAC values				CASES	TAC values				CASES	TAC values			
	q1	q2	q3	q4		q1	q2	q3	q4		q1	q2	q3	q4
34	000.000.016.016				3	008.000.000.000				1	004.004.000.016			
33	002.002.000.000				3	018.018.016.016				1	000.000.024.000			
25	000.000.000.016				3	016.000.016.016				1	001.000.000.000			
24	000.000.016.000				2	002.002.016.016				1	000.000.000.032			
21	002.000.000.000				2	000.000.000.004				1	024.016.016.000			
18	000.002.000.000				2	004.000.000.000				1	016.018.000.017			
12	000.016.000.000				2	016.000.000.016				1	000.002.016.000			
12	016.000.000.000				2	000.000.004.000				1	000.002.000.001			
11	000.000.000.001				2	000.000.064.064				1	002.000.016.016			
11	000.000.000.002				1	000.000.000.010				1	000.002.000.016			
9	000.000.001.000				1	016.016.016.024				1	072.064.064.064			
9	016.016.016.016				1	000.016.016.016				1	000.016.001.000			
8	016.016.000.000				1	016.016.016.002				1	004.004.024.024			
6	000.004.000.000				1	000.032.000.016				1	002.002.000.002			
5	000.000.002.002				1	000.002.002.000				1	026.016.000.000			
4	008.008.000.000				1	000.008.000.000				1	120.090.082.064			
4	004.004.000.000				1	016.000.016.000				1	000.000.024.024			
4	000.000.001.001				1	064.000.000.016				1	026.026.025.025			
4	000.000.002.000				1	018.000.000.000				1	018.022.000.000			

The present study used the TAC exclusively for descriptive analyses, allowing the identification of agenesis distribution patterns in the evaluated population and presenting relevant information about the regions most frequently affected by tooth absence. This approach included a detailed understanding of tooth agenesis profiles, contributing to a clinical and epidemiological assessment of the condition.

### MACHINE LEARNING MODELS

**Figure 3:**
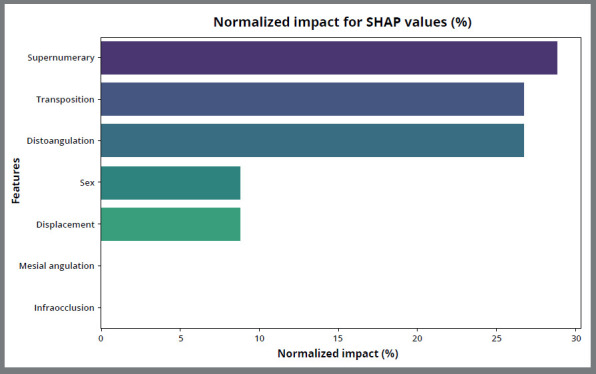
SHAP values demonstrating the normalized impact of the variables in Random Forest construction, in percentages.

Table 5 describes the metrics of the applied models. All models performed similarly, but Random Forest showed the best AUC, ACC, and F_1_ score metrics. The SHAP values showed that variables with higher relevance in this model were, in descending order: supernumerary teeth, canine transposition, and sex (Fig. 3).

**Table 5:** Performance metrics of machine learning models.

Model	AUC (95% CI)	ACC (95% CI)	PPV (95% CI)	NPV (95% CI)	F1 score (95% CI)
Decision Tree	0.5992 (0.5738 - 0.6246)	0.9415 (0.9389 - 0.9441)	0.6067 (0.4089 - 0.8044)	0.9437 (0.9414 - 0.9459)	0.1404 (0.0877 - 0.1931)
Random Forest	0.6008 (0.5738 - 0.6278)	0.9419 (0.9390 - 0.9448)	0.6367 (0.4368 - 0.8366)	0.9440 (0.9415 - 0.9466)	0.1525 (0.1037 - 0.2014)
XGBoost	0.5897 (0.5615 - 0.6180)	0.9405 (0.9373 - 0.9436)	0.5629 (0.3451 - 0.7806)	0.9429 (0.9402 - 0.9456)	0.1199 (0.0737 - 0.1660)
SVM	0.5193 (0.4839 - 0.5547)	0.9411 (0.9384 - 0.9437)	0.5900 (0.3916 - 0.7884)	0.9433 (0.9408 - 0.9457)	0.1299 (0.0789 - 0.1809)
Neural Network	0.6017 (0.5719 - 0.6315)	0.9415 (0.9385 - 0.9445)	0.6129 (0.4015 - 0.8243)	0.9440 (0.9414 - 0.9466)	0.1519 (0.1029 - 0.2009)

## DISCUSSION

### TOOTH AGENESIS AND ITS ASSOCIATION WITH OTHER DENTAL ANOMALIES

This study offers a comprehensive view of the complex associations between tooth agenesis and other dental anomalies, emphasizing the importance of early diagnosis and detailed therapeutic planning. The prevalence of non-syndromic tooth agenesis in 4,990 Brazilian patients (50% male, 50% female), aged 8 to 30 years, was 6.1%, aligning with the range reported in similar studies (3% to 13%), likely influenced by regional and national differences.^10^ ^,^ ^27^ ^,^ ^28^

Data analysis revealed a significantly higher prevalence of tooth agenesis in women (57.6%). This difference is attributed to genetic factors (e.g., greater expression of X-linked genes involved in dental development), hormonal influences (such as estrogen), and epigenetic mechanisms (e.g., sex-based differences in gene regulation), which may increase susceptibility in females.^6^ ^,^ ^9^ Early detection is therefore essential, enabling timely interventions to prevent or reduce future complications, including the need for orthodontic treatment or complex prosthetic rehabilitation.

The high prevalence of anomalies associated with tooth agenesis, such as dental transposition, deciduous molar infraocclusion, and lower second premolar distal angulation, may suggest a common genetic base for these conditions.^4^ ^,^ ^7^ ^,^ ^10^ The present study shows that approximately 15% of tooth agenesis patients presented other associated dental anomalies, especially dental transposition, palatally displaced canines, and deciduous molar infraocclusion. Therefore, the clinical management of these patients must consider the possibility of other coexistent anomalies, requiring a more comprehensive approach beyond agenesis resolution.

Regarding dental anomaly distribution between upper and lower arches and right and left sides, studies indicate that bilateral involvement patterns may be explained by the interaction of genetic factors that regulate dental development coordinately on both dental arch sides.^9^ ^,^ ^29^ ^,^ ^30^ Genes involved in dental development, such as *PAX9* and *MSX1*, have represented agenesis pattern influencers, affecting formation on both sides. The mutation of these genes may cause a symmetrical dental development failure.^4^ ^,^ ^29^ ^,^ ^31^ Thus, the present study showed higher tooth agenesis prevalence bilaterally when considering the right and left sides separately.

The literature reports that bilateral tooth agenesis may follow an autosomal dominant pattern with incomplete penetrance. That means that phenotypic expression may vary among individuals despite the presence of the mutated gene. This variability may produce an anomaly pattern that affects both dental arch sides, manifesting symmetrically.^6^ In line with the mentioned studies, this bilateral pattern may indicate an underlying genetic pattern that symmetrically affects dental development accompanied by significant clinical implications, especially in orthodontic treatment planning, in which occlusal balance is essential for therapeutic success.^32^

The found prevalence ratios indicate a strong association between agenesis and certain anomalies, with a significantly higher prevalence in patients with palatally displaced canines, dental transposition, and deciduous molar infraocclusion (Table 3). These data agree with Kantaputra et al.^33^, who show that such prevalence and associations may share etiological mechanisms, potentially from genetic mutations, especially in *PAX9, MSX1*, and *WNT10A*. These mutations may alter the regular sequence of dental formation, path, and eruption, favoring canine displacement, transposition, and anomalous dental development, possibly associated with deciduous molar infraocclusion.^33^ Lupinetti et al.^6^ reported that molar infraocclusion occurs due to a development failure in the periodontal ligament or alveolar bone. Both processes are influenced by the same genetic mutations that cause tooth agenesis and transposition due to anomalous dental germ migration during development.^6^ Growth pattern implications during development may also play a role, as tooth absence (agenesis) potentially influences the position and development of adjacent teeth. That may cause transpositions or typical eruption failures.^4^

The combined effect of multiple factors may create a favorable environment for the occurrence of several dental anomalies in the same patient. This underscores the importance of clinical monitoring for early detection, as the presence of one anomaly may indicate a predisposition to others, reinforcing the value of an integrated diagnostic approach. In this context, preventive and interceptive Orthodontics play a key role, since early identification of anomaly patterns enables timely therapeutic actions - such as intercepting eruption paths, preserving arch space, and preventing malocclusion progression. Notably, this study did not identify any cases of lower second molar mesial angulation among patients with agenesis, limiting potential comparisons. This suggests that the coexistence of these conditions is rare and that mesial angulation of the second molar is more likely influenced by mechanical or environmental factors, such as adjacent tooth eruption or lack of space in the dental arch.

### TOOTH AGENESIS AND THE TOOTH AGENESIS LABEL

The tooth agenesis label, or “code” (TAC), showed a higher prevalence in lower premolars and upper lateral incisors bilaterally. This distribution agrees with the literature, indicating these areas as most susceptible to agenesis.^4^ ^,^ ^28^ The TAC is valuable for clinical and epidemiological research, allowing tooth agenesis standardization and categorization, to improve the understanding of tooth absence patterns essential for treatment diagnosis and planning.^24^

Although TAC has aided tooth agenesis characterization, the machine learning models applied in this study did not use it directly. Its application is limited to the descriptive analysis of tooth absence patterns, providing information that may contribute to future investigations. Combining quantitative methodologies and prediction models may significantly advance the understanding of dental anomalies, as long as they are adequately integrated and validated in later studies.

### INTEGRATION WITH ARTIFICIAL INTELLIGENCE-BASED PREDICTION MODELS

Implementing the models used in the study aimed to investigate their applicability to the early diagnosis of tooth agenesis, allowing more precise preventive interventions and facilitating dental therapeutic planning. Integrating machine learning techniques into clinical analyses is a promising advance in Dentistry, allowing a more robust and customized approach to identifying dental anomalies.^34^

Recent artificial intelligence (AI) developments offer prospective opportunities to improve tooth agenesis detection and management. Random Forest was more robust regarding class imbalances than other models, probably due to the capacity to weigh samples during training. Despite the superior performance of Random Forest, it is not free of limitations. For instance, its training time and computational complexity are higher than simpler models such as the Decision Tree.^34^ ^,^ ^35^

Despite the high accuracy (94%), the model presented an F_1_ score of only 0.15 and an AUC of 0.6, indicating a low capacity to identify the minority class correctly. These indicators show that, although the model seems generally correct (high accuracy), it is not highly able to capture the relationships between variables to identify the minority class correctly.^36^ ^,^ ^37^ These findings highlight that the model, in its current form, is not adequate for clinical applications because it may fail to identify tooth agenesis patients. That may be justified by the low agenesis prevalence in the sample and the low number of prediction variables in the model.

The Random Forest analysis of variable relevance indicated the characteristics with the highest impact on tooth agenesis prediction, providing valuable insights for clinical practice. The variables related to tooth position in the present study, such as supernumerary teeth and canine transposition, showed higher contributions for model predictions. That aligns with the literature suggesting the association of tooth position alterations with congenital tooth absence.^4^ ^,^ ^10^ Conversely, variables such as sex reflect a higher prevalence of the condition in women. These findings imply that the clinical evaluation of tooth agenesis must prioritize position characteristics, especially the presence or absence of supernumerary teeth. Moreover, the impact of sex on the model reinforces the significance of epidemiological factors in tracking the condition. Although the model identified relevant variables, these findings must be validated in larger samples and different populations, to confirm its clinical applicability.

### LIMITATIONS AND FUTURE PERSPECTIVES

It is worth considering some limitations of this study. The main one is the retrospective and observational design, which may introduce selection biases and complicate result generalizations. Also, the visual evaluation of radiographs may promote diagnosis subjectivity.^18^ Another limitation is the absence of a multivariate analysis considering other possible confounding factors, such as family history and environmental influence, which might provide a more comprehensive understanding of the factors involved in tooth agenesis and their associations.

Future studies may focus on genetic analyses to identify specific genes responsible for different phenotypic manifestations and to explore the influence of environmental factors on the development of these anomalies. In addition, longitudinal research may provide insights into the progression of these conditions over time, and inform improvements in clinical management. Such studies can offer valuable data on treatment outcomes, support the evaluation of orthodontic interventions, and contribute to the development of clinical guidelines aimed at more effective and safer care for patients with non-syndromic dental anomalies.

## CONCLUSION

Tooth agenesis was identified in 6.1% of the studied population, and was more prevalent in women and younger patients. The significant association between agenesis and anomalies, such as palatally displaced canines, dental transposition, and deciduous molar infraocclusion, reinforces the hypothesis of a common genetic base for these conditions, evidencing the relevance of integrated diagnostic approaches. The predominant distribution of agenesis in lower premolars and upper lateral incisors highlights specific patterns to be considered in clinical planning. Random Forest was the most effective model to predict tooth agenesis. Its robustness and capacity to generalize complex patterns among the variables highlighted it as a promising choice for future studies and practical dental applications. Further investigations involving genetic analyses and longitudinal assessments might deepen the knowledge of underlying mechanisms and their clinical implications.

## Data Availability

Due to the sensitive nature of the data and ethical restrictions, the datasets supporting this study are not publicly available.
